# Prevalence of ear disease in cats undergoing cone beam computed tomography for dental procedures

**DOI:** 10.3389/fvets.2025.1553585

**Published:** 2025-03-14

**Authors:** Hannah F. Boothe, Mary Krakowski Volker, Jennifer Tjepkema, Adrien-Maxence Hespel

**Affiliations:** ^1^Animal Dental Center, Annapolis, MD, United States; ^2^Animal Dental Center, York, PA, United States; ^3^Perth Veterinary Specialists, Osborne Park, WA, Australia

**Keywords:** cone beam computed tomography, cat, ear disease, dentistry, feline skull

## Abstract

**Introduction:**

In this prospective, cross-sectional study, 303 feline patients were evaluated via cone beam computed tomography (CBCT) for evidence of incidental ear disease during a dental procedure.

**Methods:**

All feline patients over one year of age presenting to a private practice veterinary dentistry and oral surgery specialty clinic underwent CBCT imaging which included the oral cavity and ear canals. The following were recorded if present: periodontal disease, tooth resorption and/or ear disease.

**Results:**

Ear disease was diagnosed as an incidental finding in 41.4% of cats that were imaged; this is higher than previously reported prevalence studies of the general feline population ranging from 2-19%. Periodontal disease and tooth resorption were not significantly associated with ear disease. However, cats less than or equal to 10 pounds were found to be at higher risk of ear disease.

**Discussion:**

This study highlights the importance of advanced imaging in the diagnosis of not only dental disease but also ear disease in cats.

## Introduction

Cats are notorious for subclinical disease, hiding signs until the condition has progressed considerably. Ear disease in cats can be especially difficult to diagnose, either due to vague clinical signs, limited tolerance of a thorough veterinary examination, or anatomic conformation (1). The anatomy of the skull is complex and there is particular interest in having better diagnostic modalities available to clinicians to understand the association of sinuses, ear canals and their relationship with the oral cavity in feline patients.

Given the complex skull structure and close location between the oral cavity and ear canal system in cats, it is reasonable to question the pathophysiology of each in relation to each other. Oral and sinonasal relationships have been found in veterinary canine and human medicine. In 2015, Stepaniuk and Gingerich found the source of inflammatory rhinitis was determined to be odontogenic in 55% of canine cases (2). Within human medicine, it was found that in a sinus with over two-thirds opacified by fluid, 79% of patients had an identifiable dental source visualized on computed tomography (CT) (3). Also in humans, periodontal bone loss was found to be associated with maxillary sinusitis as diagnosed by CBCT (4).

The advancement of diagnostic imaging in the form of CT, magnetic resonance imaging (MRI), and CBCT has allowed for further understanding of these relationships and their potential sequelae. A 2003 study in dogs compared MRI and CT in which CT was found to provide advanced bone detail while MRI was superior for visualization of soft tissue structures (5). Computed tomography in cats has been shown to accurately identify multiple anatomical structures of the head including the nasolacrimal system, nasal cavity and paranasal sinuses, and the infraorbital canal (1, 6–9). These advanced imaging modalities have proven to be effective diagnostic tools in veterinary patients. Although CT and MRI have been traditionally used, there is a paucity of studies utilizing CBCT as the primary diagnostic imaging source for evaluation of otic disease in cats.

Comparisons of both CT and CBCT have shown improvements in the diagnosis of dental disease with CBCT. Advantages of CBCT include higher resolution, specifically of bone anatomy, with lower radiation as well as lower equipment cost and increased speed of the diagnostic scan. Disadvantages include more diverse scatter direction and decreased image quality for soft tissue structures compared to conventional CT methods (4, 10–12). CBCT images were found to be superior in image quality compared to 64-multidetector row CT in various fields of view and six defined dentoalveolar anatomic structures in dogs (10). Two studies have proven the usefulness of CBCT for the diagnosis of dental abnormalities and anatomic structures in small animals (10, 44). Specifically in cats, CBCT yielded significant improvements in identification of both anatomic dental structures and dentoalveolar lesions in multiple categories (13, 14). Similar findings were found in small to medium sized brachycephalic dogs further assessing the increased diagnostic yield of multiple oral anatomic landmarks and dental disorders (15, 16).

There are a few studies whose focus was to evaluate the prevalence of ear disease utilizing CT/CBCT in both human and veterinary medicine. In humans, CBCT has been used for middle and inner ear implant evaluation (12). In dogs, these studies include CT scans that have allowed for anatomic descriptions of the internal ear canals and surrounding head structures (17–20). Middle ear disease was retrospectively diagnosed in cats via CT finding that 34% of cats did not have a primary complaint of clinical ear disease (21). No such prevalence or clinical assessment studies have been published yet with inclusion of cone beam computed tomography. Given that a CBCT scan includes the sinonasal cavities, oral cavity, and ear canal system, it is of ethical importance for the practitioner to report on the entire set of results, even beyond the primary focus of the oral cavity.

The primary objective of the study was to evaluate the prevalence of ear disease in cats undergoing CBCT imaging during dental procedures. The secondary objective was to evaluate associations between periodontal disease or tooth resorption with ear disease. Finally, tertiary objectives included evaluation of any potential associations between seasons, age, sex, and weight with the outcomes of periodontal disease, tooth resorption, and ear disease. We hypothesized that prevalence of ear disease on CBCT imaging for dental procedures would match the overall population studies in cats. We questioned if there was a correlation between periodontal disease and/or tooth resorption in these cases.

## Materials and methods

This was a prospective, cross-sectional study of 303 feline patients. Analysis of data management, descriptive statistics, and were performed using R language^1^. Statistical models were performed using STATA^2^.

A CBCT scan was performed on all anesthetized feline patients at the Animal Dental Center in Columbia, Maryland and York, Pennsylvania. Patients under 1 year of age were excluded due to their decreased prevalence of periodontal disease and tooth resorption (22, 23).

Clients signed a media and photography release authorizing the use and release of all diagnostic imaging/data obtained from the procedure for future publications and clinical studies. Prior to the appointment, a patient intake form was provided to the client with questions relating to potential clinical signs of ear disease.

The client was provided either a paper version or electronic drop-down of “yes” or “no” for the following questions:

1. Has your pet been diagnosed with any form of ear disease in the past 2 weeks?
2. In the last 2 weeks, have there been any signs of ear problems including but not limited to head shaking, rubbing, or scratching at the ears, and/or ear discharge?

### Exclusions

A total of 311 cats were scanned over a year (10/5/2022 to 10/5/2023). Eight cats were excluded for the following reasons: two were initially scanned prior to the start (i.e., follow-up procedures), one owner declined to be part of the study, four patients were not scanned due to technical machine errors, and one patient did not have a scan performed. This allowed for a total of 303 feline patients to be included in the study. A full year of data collection was chosen to evaluate if there was a seasonal association (24).

### Data review methods

Patients were placed under general anesthesia and complete anesthetic monitoring was performed for each patient. A CBCT scan^3^ was performed during a comprehensive oral health assessment and treatment. The entire skull, including dentition and ear canals, were included in the scans. CBCT scans were reviewed at a later date through third-party viewing software^4^ at 0.1 mm slice intervals. Periodontal disease and tooth resorption was evaluated and data input performed by two Board Certified Veterinary Dentists™ and one second/third year resident in an AVDC-approved veterinary dentistry and oral surgery training program. Ear disease diagnoses and data input was performed by a board-certified veterinary radiologist. Variables and associated categories are described below.

### Ear disease variables and categorization

Multiple variables were evaluated for the study. Primary objectives included the frequency and number of patients with ear disease as a “yes” or “no.” Ear disease was further classified by location of “left,” “right,” “externa,” “media,” and “interna.”

Definitions of and criteria for ear disease were as follows (18):

1. Mass or attenuating material within the external ear canal.
2. Ear canal mineralization or stenosis up to the level of the tympanic membrane.
3. Abnormalities of the tympanic bulla including wall thickening, lysis, or mineralized bodies.
4. Mineralization, attenuating material, or periosteal proliferation of the temporal bone, cochlea, the external and internal acoustic meatus, and/or the semicircular canals, including the tympanic cavity.

**Figure 1:**
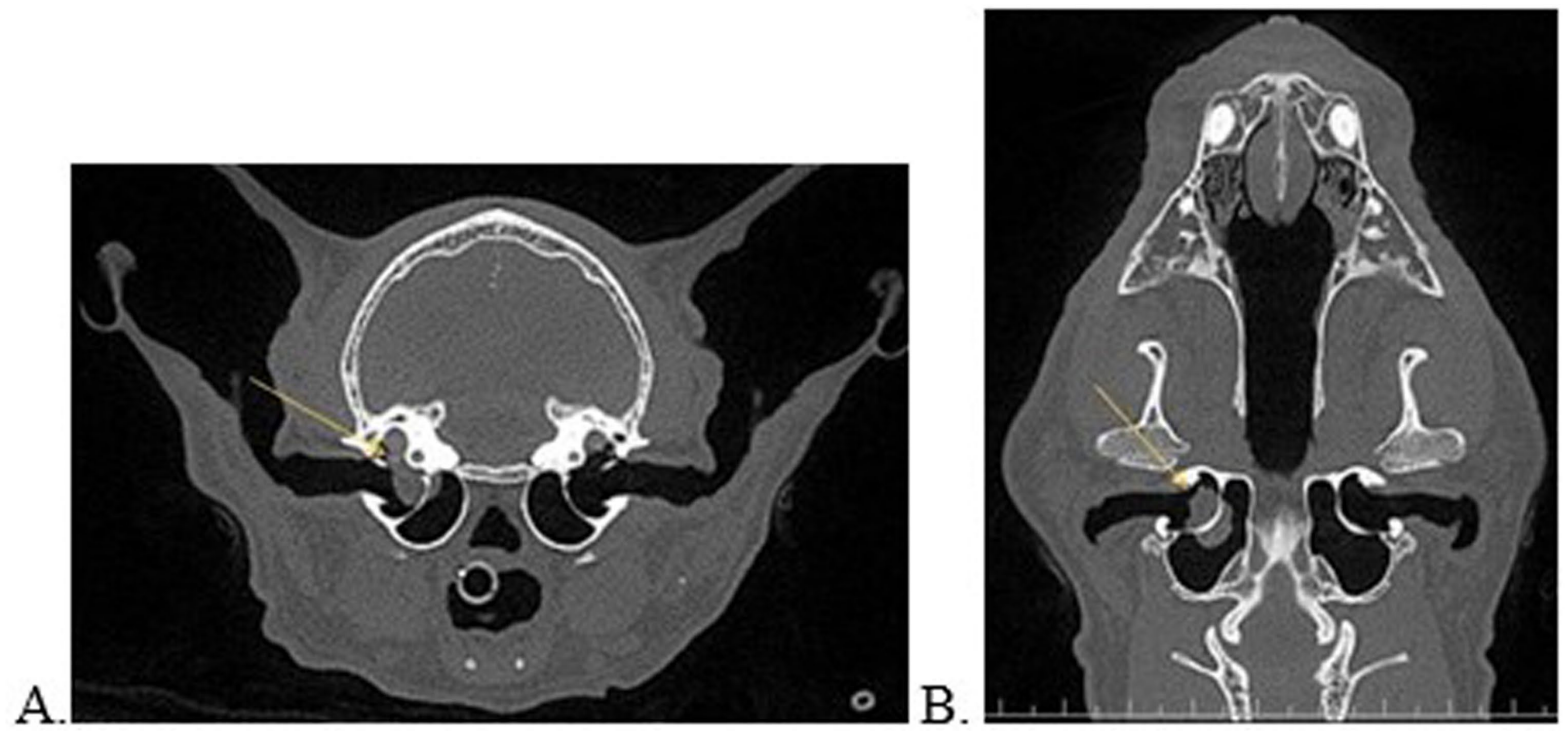
Transverse **(A)** and dorsal **(B)** multiplanar reconstructed views of a 17-year-old, asymptomatic cat with consolidated material on either side of the bony septum of the middle ear and concurrent extension into the inner ear (**A,B**, yellow arrow).

**Figure 2:**
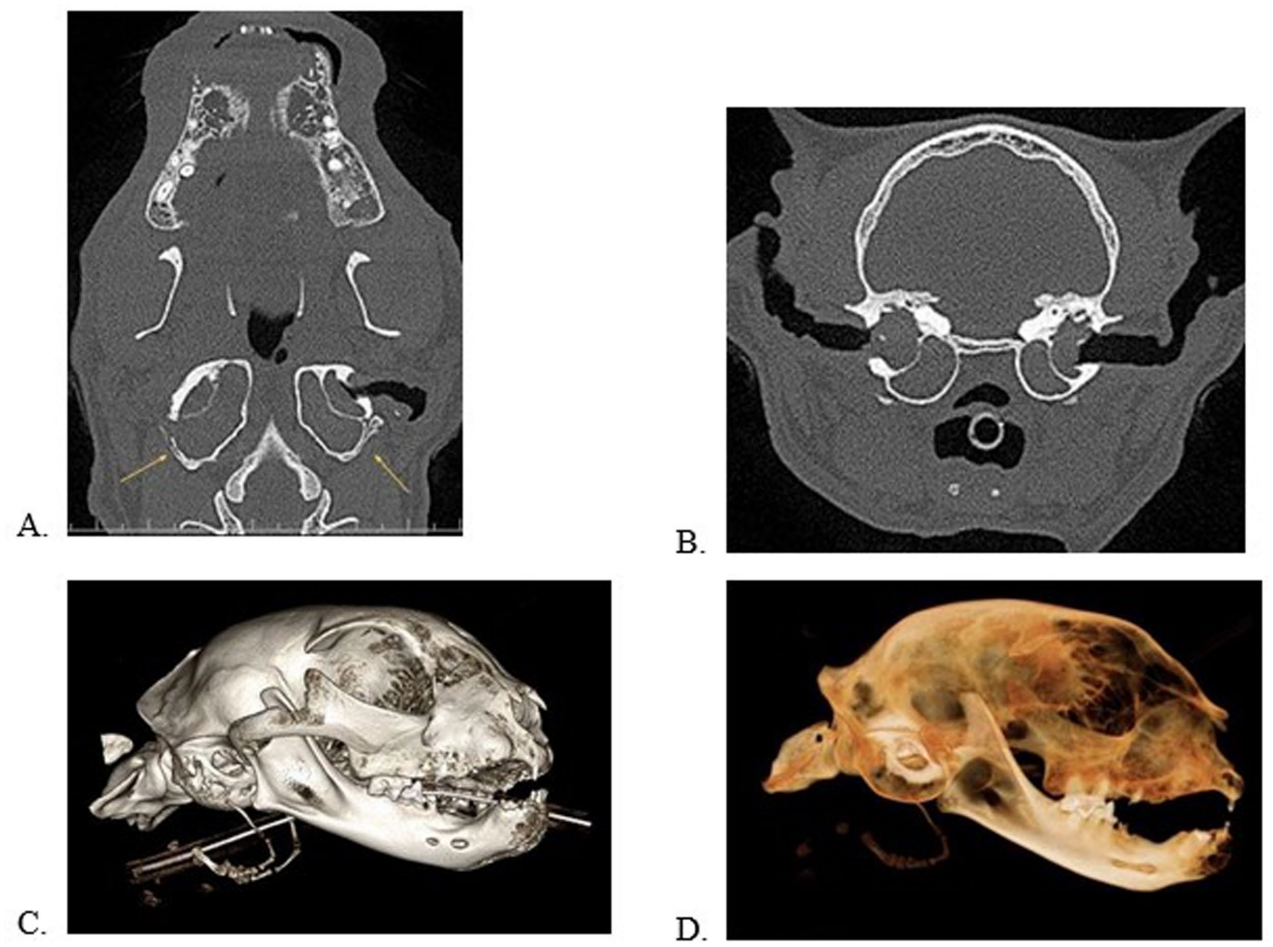
Multiplanar views in the dorsal **(A)** and transverse **(B)** planes and 3D volume renderings in a bone **(C)** and dental **(D)** algorithm of a cat with bilateral, external, middle, and internal ear disease with clinical signs. Note the deformation/remodeling of the tympanic bulla (**A**, yellow arrows). This patient has significant alveolar bone expansion, periodontal disease, and tooth resorption.

**Figure 3:**
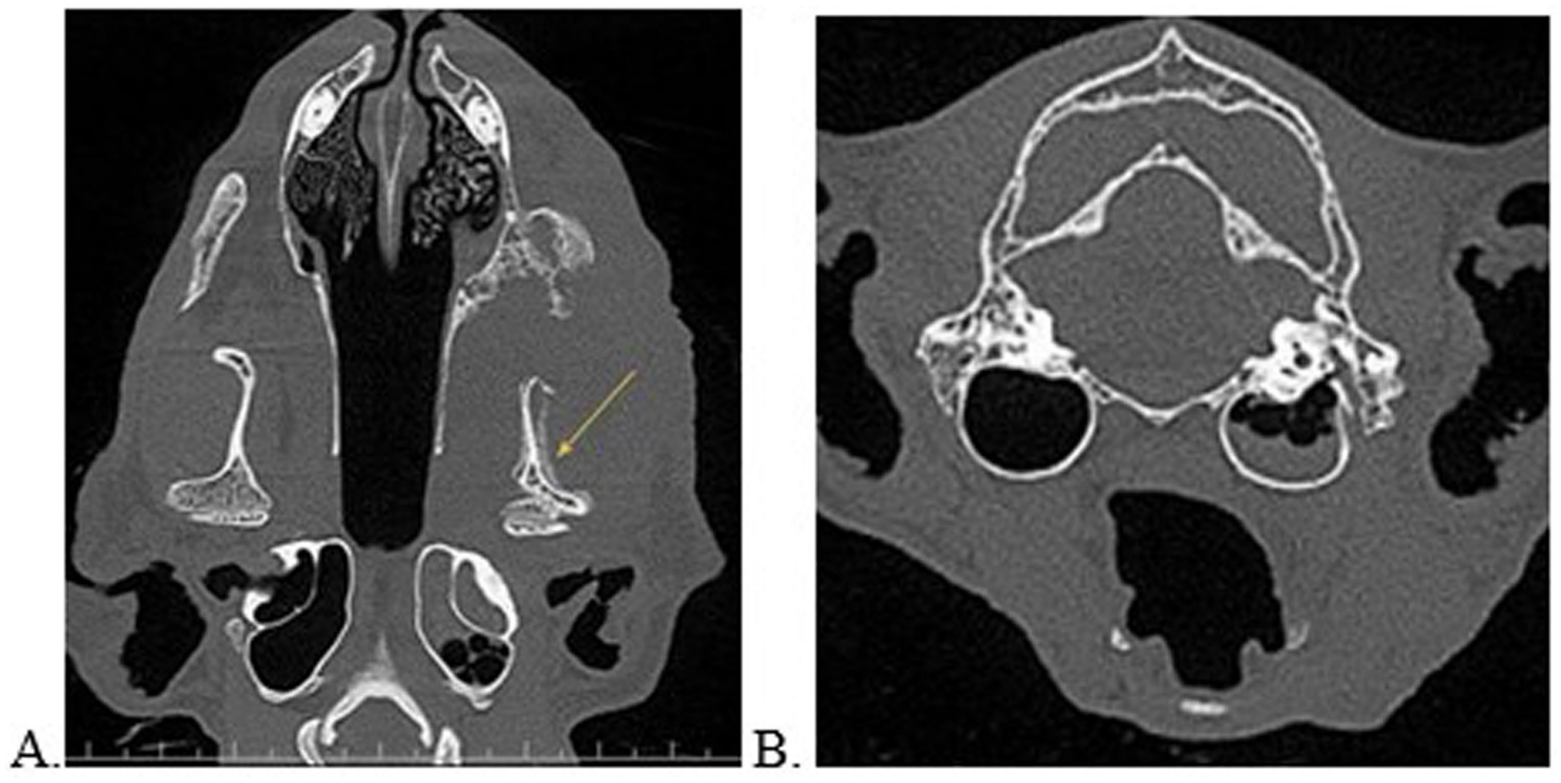
Multiplanar reconstructed views in the dorsal **(A)** and transverse **(B)** planes of a cat with left sided oral and ear disease. Note the loculated regions of hypoattenuation against a solidly hyperattenuated tympanic bulla consistent with an air-fluid interface. This patient was concurrently diagnosed with tooth resorption, periodontal disease, left sided soft tissue swelling, and severe periosteal reaction of the left mandibular bone extending to the temporomandibular joint (yellow arrow). There was high suspicion for a neoplastic source. This patient was unable to open the mouth and the owner answered “yes” to noted aural clinical signs.

**Figure 4:**
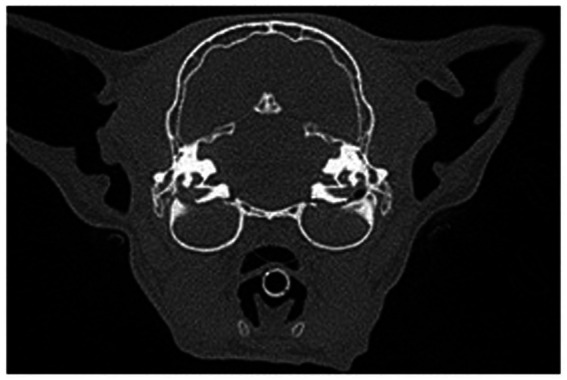
Transverse view in a multiplanar reconstruction of a feline patient with bilateral ear disease of both the middle and internal ear anatomy exhibited by complete attenuation of the tympanic bulla.

**Figure 5:**
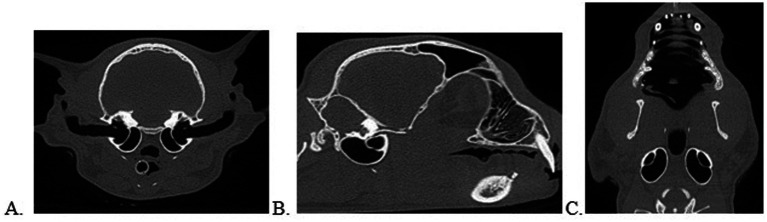
Normal ear anatomy as viewed on CBCT multiplanar reconstruction in the transverse **(A)**, sagittal **(B)**, and dorsal **(C)** views.

Figures 1–4 exemplify patients in the current study with ear disease of various forms based on the above definitions. For reference of normal anatomy, Figure 5 is provided.

### Additional variables and categorization

Variables assessed based on the secondary and tertiary objectives included tooth resorption, periodontal disease, season, age, sex, weight. Anatomical locations for evaluation of tooth resorption and periodontal disease were divided into two variables: rostral (incisors and canines) and caudal (premolars and molars). Both periodontal disease and tooth resorption were evaluated by diagnostic imaging only; clinical examination was not performed. Tooth resorption was further categorized by type (0, 1, 2, 3) based on AVDC definitions (25). If more than one type of tooth resorption was diagnosed within a variable, it was defined as “multiple.” Periodontal disease was categorized into rostral and caudal variables with further identification of the stage (0, 2, 3, 4) based on AVDC definitions (25). Stage 1 was excluded as this is a clinical only diagnosis. For statistical analysis of tooth resorption, categories of “3” and “multiple” types were combined.

Seasons were categorized based on the date of the procedure. Fall included September 1 to November 30, Winter from December 1 to February 28, Spring from March 1 to May 31, and Summer from June 1 to August 31.

Age was categorized as 1–6 years, 7–10 years, and greater than 10 years old based on previously published age categories (26, 27). Weight was categorized as less than or equal to 10 pounds and greater than 10 pounds. Skull conformation and body condition score were not recorded.

### Statistical analysis

The objective results were estimated via an intercept-only generalized linear mixed-effect model (GLMM) with a binomial distribution. Since data collection occurred over two locations, a random intercept for clinic (*n*=2) was included to account for non-independence.

## Results

### Ear disease, periodontal disease, tooth resorption

The overall prevalence of ear disease in cats undergoing CBCT imaging was 41.4% (95% CI = 31.4 51.5%). One hundred and twenty-two cats (122) were positive on imaging for ear disease. Forty-two (42) cats were diagnosed with unilateral ear disease while 80 had bilateral ear disease. There was no significant difference between the side of ear disease diagnosis (left versus right).

Periodontal disease, rostral and caudal, was diagnosed in 238 patients. Tooth resorption, rostral and caudal, was diagnosed in 171 patients. Prevalence of periodontal disease was 78% and prevalence of tooth resorption was 56%.

There was no statistically significant association between periodontal disease or tooth resorption with ear disease diagnosed on CBCT. Season was not significantly associated with ear disease (*p* = 0.08). Patients weighing less than or equal to 10 pounds had a significantly higher prevalence of ear disease (47.8, 95% CI = 36.2–59.4%) than those weighing greater than 10 pounds (35.9, 95% CI = 25.3–46.4%) holding sex and age constant (*p* = 0.04). Age and sex were not significantly associated with ear disease (*p* = 0.09 and *p* = 0.13, respectively).

### Other factors

Between the two locations, 137 cats were evaluated in the York, Pennsylvania office and 166 cats evaluated in the Columbia, Maryland office.

Age range included 1–18 years with 127 cats age 1–6 years, 85 cats age 7–10 years, and 91 cats greater than 10 years old. Age was significantly associated with rostral tooth resorption score (*p* = 0.02). Cats 7–10 years old and older than 10 years of age were 2.2 times (*p* = 0.02) and 2.7 times more likely to have a rostral tooth resorption score of “2,” respectively (compared with rostral tooth resorption score of “0”), than 1–6-year-old cats (*p* < 0.01). Cats 7–10 years old were 3.4 times (*p* = 0.03) more likely to have a rostral tooth resorption score of “multiple” (compared with rostral tooth resorption score of “0”) than 1–6-year-old cats. Age (*p* = 0.06), weight (*p* = 0.54), and sex (*p* = 0.39) were not significantly associated with caudal tooth resorption score. Age (*p* = 0.09), weight (*p* = 0.47), and sex (*p* = 0.22) were not significantly associated with rostral periodontal disease.

Weight range was 4.5–27 pounds with 137 cats less than 10 pounds and 166 greater than 10 pounds. Median weight was 10.2 pounds and average weight was 10.8 lbs. Female spayed status included 140 patients while 163 patients were male neutered. No intact patients were present.

Age was significantly associated with caudal periodontal disease (*p* = 0.05). However, after controlling for multiple comparisons using a Bonferroni adjustment, no pairwise comparisons were statistically significant (*p* > 0.1).

Although season was not significantly associated with ear disease (*p* = 0.08), the prevalence of ear disease was the highest in the summer (50.8%).

### Client survey

Client questionnaire answers were excluded from statistical analysis due to multiple uncontrolled client factors such as: incomplete form submission, inappropriate time completion of the form (weeks before or after surgery), and inconsistency in answers (selecting “no” for ear disease then discussing ear disease later in the form). From the included raw data, 9 clients did not answer the questions, 257 responded “no,” and 37 responded “yes.” One hundred and eight (36%) clients answered “no” in regards to clinical signs with the patient subsequently diagnosed with ear disease upon imaging evaluation. Fifteen clients (<1%) answered “yes” to clinical signs with a subsequent positive diagnosis of ear disease. Twenty-two (<1%) clients answered “yes” to clinical signs with no ear disease diagnosed on imaging.

## Discussion

Our study found that 41% of cats undergoing CBCT evaluation for dental procedure had incidental ear disease diagnosed by CBCT. Overall, periodontal disease and tooth were resorption were not significantly associated with ear disease diagnosed on CBCT (Tables 1, 2). Caudal periodontal disease and rostral tooth resorption were more prevalent in patients 7–10 years old compared to 1–6 years old. The older cats (7-10 years old and older than 10) were more likely to have Type 2 resorption or multiple types of tooth resorption compared to 1-6 year olds. In addition, our study was consistent with previous reports with older cats exhibiting a higher incidence of periodontal disease and tooth resorption (22, 28, 29).

**Table 1:** Frequency table of the number and percentage of cats diagnosed with ear disease while undergoing CBCT procedures by and across rostral and caudal periodontal disease score.

Ear disease diagnosed	Periodontal disease score				
	*N* (%)				Total
	Score 0	Score 2	Score 3	Score 4	
Rostral					
Yes	34 (27.6)	28 (22.8)	21 (17.1)	40 (32.5)	123 (100)
No	63 (35.0)	38 (21.1)	24 (13.3)	55 (30.6)	180 (100)
Total	97 (32.0)	66 (21.8)	45 (14.8)	95 (31.3)	303 (100)
Caudal					
Yes	40 (32.5)	12 (9.8)	11 (8.9)	60 (48.8)	123 (100)
No	69 (38.3)	20 (11.1)	19 (10.6)	72 (40.0)	180 (100)
Total	109 (36.0)	32 (10.6)	30 (9.9)	132 (43.6)	303 (100)

**Table 2:** Frequency table of the number and percentage of cats diagnosed with ear disease while undergoing CBCT procedures by and across rostral and caudal tooth resorption score.

Ear disease diagnosed	Tooth resorption score					
	*N* (%)					Total
	Score 0	Score 1	Score 2	Score 3	Multiple	
Rostral						
Yes	62 (48.8)	22 (17.9)	31 (25.2)	0 (0.0)	10 (8.1)	123 (100)
No	81 (45.6)	28 (15.6)	58 (32.2)	1 (0.6)	11 (6.1)	180 (100)
Total	143 (47.2)	50 (16.5)	89 (29.4)	1 (0.3)	21 (6.9)	303 (100)
Caudal						
Yes	33 (26.8)	40 (32.5)	17 (13.8)	1 (0.8)	32 (26.0)	123 (100)
No	42 (23.3)	47 (26.1)	48 (26.7)	1 (0.6)	42 (23.3)	180 (100)
Total	75 (24.7)	87 (28.7)	65 (21.4)	2 (0.7)	74 (24.4)	303 (100)

This study’s secondary aim was to determine if periodontal disease and tooth resorption in cats is associated with ear disease. Various causes of ear disease have been proposed ranging from narrow ear canals to soft palate abnormalities in both dogs and cats (30, 31). Nemec et al. in the Veterinary Surgery journal found the most common craniomaxillofacial abnormality in dogs with congenital palate defects to be of the tympanic bullae with most being hypoplastic. Follow up of these dogs found minimal observable clinical signs consistent with otitis media (32). A population study of stray cats in Italy found that urban location and winter season were each a risk factor in diagnosis of Malassezia species otitis based on cytology (33). Interestingly, although not statistically significant, in the present study the prevalence of ear disease was highest in the summer. Polyps and extension of ear mites through the tympanic bulla have been proposed as a factor in cases with otitis media (24). Ultimately, the origin of ear disease is likely multifactorial. In various population studies in dogs, an overall incidence rate of 7–16% ear disease has been reported, whereas in cats, ear disease population reports range from 2 to 19% (33–38). Our higher prevalence of 41% is attributed to the increased diagnostic value of CBCT by allowing visualization of internal structures including the middle and inner ear canal system.

Although a significant association between periodontal disease and/or tooth resorption and ear disease was not found in our study, findings might have been different if assessing the general population. Given the elusive nature of clinical signs in cats and the higher-than-expected diagnosis of ear disease, we suspect a positive correlation remains possible. The population in this study was pooled from a dental referral specialty practice, with the majority of cases having oral disease. Our results of periodontal disease at 78% is consistent a previous radiographic imaging study of 72% periodontitis (23). Similarly, our results of prevalence 56% tooth resorption are consistent with two previous radiographic imaging studies of 60.8 and 66% (39, 40). While many of these prevalence studies have been performed on dental patients, one recent random population study in Denmark found a prevalence of 40% tooth resorption as diagnosed via oral examination, CBCT, and dental radiography. In addition, this study found CBCT to have >99% sensitivity and specificity for diagnosis of tooth resorption (41). As CBCT imaging for dental procedures increasingly becomes standard of care within specialty dentistry practices, a different relationship may be determined. While an exclusively dentistry practice is unlikely to perform the aural treatments, referral to a dermatologist or continued monitoring of clinical signs of ear disease is prudent based on abnormal clinical diagnoses.

Neither age nor sex were significantly associated with ear disease. However, weight was considered significant. Cats weighing less than or equal to 10 pounds had a significantly higher prevalence of ear disease than those weighing more than 10 pounds. It is important to note that body condition score was not evaluated in this study therefore underweight or overweight status may have influenced this result. In addition, skull conformation was not recorded. However, Davis et al. demonstrated via CT analysis a smaller infraorbital canal in brachycephalic cats with the eye nearer to the maxillary foramen (1). Therefore, it is not unreasonable to hypothesize a closer proximity of the ear anatomy to surrounding oral structures in smaller cats. Further characterization of the feline skull conformation could potentially determine the validity of this hypothesis.

The client questionnaire was excluded from statistical analysis primarily due to the difficulties within public surveying. While the questions were formulated to allow for only “yes” or “no” answer choices, variation and uncontrolled factors ensued. In one specific example, a client answered “no” but wrote later in the comments the patient had a recent diagnosis of ear mites. The obstacles encountered in this survey highlight the enigmatic nature of evaluation of clinical signs of disease in cats. Our study found that over one-third of cats had diagnostic imaging criteria consistent with ear disease while 85% of clients indicated an answer of “no” for positive clinical signs. Only 5% of clients answered “yes” to recent clinical signs with ear disease diagnosed at imaging review. 35% indicated no clinical signs with their pet diagnosed with ear disease, further stressing the subtle nature of clinical signs and difficulty for detection by owners. Interestingly, 7% of clients answered “yes” on the questionnaire with no imaging diagnosis of ear disease. It is postulated these patients had not yet developed progressive disease that would be visualized on CBCT.

Advanced ear disease in cats can progress to vestibular signs, Horner’s syndrome, and pain. Within our study population, only 12% of clients answered “yes” to the clinical questionnaire. One previous necropsy study found a result of 10% of patients with clinical signs of vestibular disease, one with Horner’s syndrome prior to death, and 1.7% with non-neoplastic middle ear disease (42). Another histologic study found that 48% of cats had histologic evidence of middle ear disease and only 34% showed gross evidence of lesions (43). It is interesting that the histologic study results closely resembled our result of 41% of imaging abnormalities within the ear anatomy. Thus, when a client perceives clinical signs of feline aural abnormalities, a thorough physical exam followed by appropriate diagnostics and treatment are recommended. This survey further stresses the importance of periodic veterinary examinations for the feline patient and need for advanced imaging in many cases.

In conclusion, 41% of feline patients presenting for dental procedures exhibited CBCT changes consistent with ear disease. Limitations to this study include the absence of clinical gross visualization of the ear canals as well as lack of cytology or culture sampling. Myringotomy culture and cytology sampling may allow differentiation beyond the broad classification of “ear disease.” In addition, the ear anatomy in this study was evaluated by a single board-certified veterinary radiologist compared to the dental disease evaluation by multiple persons. It is to be noted that in many dentistry centers, a radiology specialist may not be readily available or may not be tasked to evaluate CBCT when looking at dental disease. We therefore believe that review by board-certified veterinary radiologist was able to provide a high level of accuracy and quality to this study. Future studies may consider evaluation by multiple radiologists for a more comparative understanding. Lastly, evaluation of left versus right dental disease, or maxillary versus mandibular, in comparison to ipsilateral or contralateral ear disease may have provided a better anatomic understanding of each disease’s pathophysiology. While this study was not able to assess the sequelae of positive clinical signs and ear disease diagnosis on CBCT, continued studies on the relevance of these imaging changes are warranted.

## Data Availability

The raw data supporting the conclusions of this article will be made available by the authors, without undue reservation.

## Footnotes

^1^Version 4.3.3 R Core Team 2024, R: A Language and Environment for Statistical Computing, R Foundation for Statistical Computing, https://www.R-project.org/, Vienna, Austria.

^2^StataCorp. 2023, StataCorp LLC, Stata Statistical Software: Release 18. https://www.stata.com/, College Station, TX.

^3^VetCAT and VetCAT IQ, Xoran, https://xorantech.com/products, Ann Arbor, MI.

^4^Invivo 7, Osteoid, https://www.osteoidinc.com/invivo-7/, Santa Clara, CA.

## References

[1] Davis LV Hoyer NK Boscan P Rao S Rawlinson JE. Computed tomography analysis of the feline infraorbital foramen and canal. Front Vet Sci. (2021) 7:619248. doi: 10.3389/fvets.2020.619248, PMID: 33585606 PMC7873595

[2] Stepaniuk KS Gingerich W. Suspect odontogenic infection etiology for canine Lymphoplasmacytic rhinitis. J Vet Dent. (2015) 32:22–9. doi: 10.1177/089875641503200103 26197687

[3] Bomeli SR Branstetter BF 4th Ferguson BJ. Frequency of a dental source for acute maxillary sinusitis. Laryngoscope. (2009) 119:580–4. doi: 10.1002/lary.20095, PMID: 19160401

[4] Phothikhun S Suphanantachat S Chuenchompoonut V Nisapakultorn K. Cone-beam computed tomographic evidence of the association between periodontal bone loss and mucosal thickening of the maxillary sinus. J Periodontol. (2012) 83:557–64. doi: 10.1902/jop.2011.110376, PMID: 21910593

[5] De Rycke LM Saunders JH Gielen IM van Bree HJ Simoens PJ. Magnetic resonance imaging, computed tomography, and cross-sectional views of the anatomy of normal nasal cavities and paranasal sinuses in mesaticephalic dogs. Am J Vet Res. (2003) 64:1093–8. doi: 10.2460/ajvr.2003.64.1093, PMID: 13677385

[6] Schlueter C Budras KD Ludewig E Mayrhofer E Koenig HE Walter A. Brachycephalic feline noses: CT and anatomical study of the relationship between head conformation and the nasolacrimal drainage system. J Feline Med Surg. (2009) 11:891–900. doi: 10.1016/j.jfms.2009.09.010, PMID: 19857852 PMC11383020

[7] Losonsky JM Abbott LC Kuriashkin IV. Computed tomography of the normal feline nasal cavity and paranasal sinuses. Vet Radiol Ultrasound. (1997) 38:251–8. doi: 10.1111/j.1740-8261.1997.tb00851.x, PMID: 9262679

[8] Nöller C Henninger W Grönemeyer DH Hirschberg RM Budras KD. Computed tomography- anatomy of the normal feline nasolacrimal drainage system. Vet Radiol Ultrasound. (2006) 47:53–60. doi: 10.1111/j.1740-8261.2005.00105.x, PMID: 16429985

[9] Tromblee TC Jones JC Etue AE Forrester SD. Association between clinical characteristics, computed tomography characteristics, and histologic diagnosis for cats with sinonasal disease. Vet Radiol Ultrasound. (2006) 47:241–8. doi: 10.1111/j.1740-8261.2006.00134.x, PMID: 16700173

[10] Soukup JW Drees R Koenig LJ Snyder CJ Hetzel S Miles CR. Comparison of the diagnostic image quality of the canine maxillary dentoalveolar structures obtained by cone beam computed tomography and 64-multidetector row computed tomography. J Vet Dent. (2015) 32:80–6. doi: 10.1177/089875641503200201, Erratum in: J Vet Dent. 2023;40(3):264. doi: 10.1177/08987564221146049 26415384 PMC5140098

[11] Miracle AC Mukherji SK. Conebeam CT of the head and neck, part 1: physical principles. AJNR Am J Neuroradiol. (2009) 30:1088–95. doi: 10.3174/ajnr.A1653, PMID: 19439484 PMC7051341

[12] Miracle AC Mukherji SK. Conebeam CT of the head and neck, part 2: clinical applications. AJNR Am J Neuroradiol. (2009) 30:1285–92. doi: 10.3174/ajnr.A1654, PMID: 19461061 PMC7051564

[13] Heney CM Arzi B Kass PH Hatcher DC Verstraete FJM. Diagnostic yield of dental radiography and cone-beam computed tomography for the identification of anatomic structures in cats. Front Vet Sci. (2019) 6:58. doi: 10.3389/fvets.2019.00058, PMID: 30873423 PMC6404553

[14] Heney CM Arzi B Kass PH Hatcher DC Verstraete FJM. The diagnostic yield of dental radiography and cone-beam computed tomography for the identification of dentoalveolar lesions in cats. Front Vet Sci. (2019) 6:42. doi: 10.3389/fvets.2019.00042, PMID: 30847347 PMC6393352

[15] Döring S Arzi B Barich CR Hatcher DC Kass PH Verstraete FJM. Evaluation of the diagnostic yield of dental radiography and cone-beam computed tomography for the identification of anatomic landmarks in small to medium-sized brachycephalic dogs. Am J Vet Res. (2018) 79:54–61. doi: 10.2460/ajvr.79.1.54, PMID: 29287153

[16] Döring S Arzi B Hatcher DC Kass PH Verstraete FJM. Evaluation of the diagnostic yield of dental radiography and cone-beam computed tomography for the identification of dental disorders in small to medium-sized brachycephalic dogs. Am J Vet Res. (2018) 79:62–72. doi: 10.2460/ajvr.79.1.62, PMID: 29287156

[17] Love NE Kramer RW Spodnick GJ Thrall DE. Radiographic and computed tomographic evaluation of otitis media in the dog. Vet Radiol Ultrasound. (1995) 36:375–9. doi: 10.1111/j.1740-8261.1995.tb00279.x

[18] Foster A Morandi F May E. Prevalence of ear disease in dogs undergoing multidetector thin-slice computed tomography of the head. Vet Radiol Ultrasound. (2015) 56:18–24. doi: 10.1111/vru.12180, PMID: 25046431

[19] Russo M Covelli EM Meomartino L Lamb CR Brunetti A. Computed tomographic anatomy of the canine inner and middle ear. Vet Radiol Ultrasound. (2002) 43:22–6. doi: 10.1111/j.1740-8261.2002.tb00437.x 11866039

[20] Probst A Kneissl S. Computed tomographic anatomy of the canine temporal bone. Anat Histol Embryol. (2006) 35:19–22. doi: 10.1111/j.1439-0264.2005.00631.x, PMID: 16433668

[21] Shanaman M Seiler G Holt DE. Prevalence of clinical and subclinical middle ear disease in cats undergoing computed tomographic scans of the head. Vet Radiol Ultrasound. (2012) 53:76–9. doi: 10.1111/j.1740-8261.2011.01873.x 22092494

[22] Vapalahti K Virtala AM Joensuu TA Tiira K Tähtinen J Lohi H. Health and behavioral survey of over 8000 Finnish cats. Front Vet Sci. (2016) 3:70. doi: 10.3389/fvets.2016.00070, PMID: 27622188 PMC5002895

[23] Lommer M Verstraete FJ. Radiographic patterns of periodontitis in cats: 147 cases (1998–1999). JAm Vet Med Assoc. (2001) 218:230–4. doi: 10.2460/javma.2001.218.230, PMID: 11195829

[24] Gotthelf LN. Diagnosis and treatment of otitis media in dogs and cats. Vet Clin North Am Small Anim Pract. (2004) 34:469–87. doi: 10.1016/j.cvsm.2003.10.007, PMID: 15062620

[25] American Veterinary Dental College. (2005) AVDC® Nomenclature. Available at: https://avdc.org/avdc-nomenclature/ (Accessed October 27, 2024)

[26] Bellows J Center S Daristotle L Estrada AH Flickinger EA Horwitz DF. Evaluating aging in cats: how to determine what is healthy and what is disease. J Feline Med Surg. (2016) 18:551–70. doi: 10.1177/1098612X16649525, PMID: 27370393 PMC10816674

[27] Quimby J Gowland S Carney HC DePorter T Plummer P Westropp J. 2021 AAHA/AAFP Feline Life Stage Guidelines. J Feline Med Surg. (2021) 23:211–33. doi: 10.1177/1098612X21993657. Erratum in: J Feline Med Surg. 2021;23(8):NP3. doi:10.1177/1098612X211024041, PMID: 33627003 PMC10812130

[28] Mestrinho LA Runhau J Bragança M Niza MM. Risk assessment of feline tooth resorption: a Portuguese clinical case control study. J Vet Dent. (2013) 30:78–83. doi: 10.1177/089875641303000202, PMID: 24006716

[29] Mestrinho LA Louro JM Gordo IS Niza MMRE Requicha JF Force JG. Oral and dental anomalies in purebred, brachycephalic Persian and exotic cats. J Am Vet Med Assoc. (2018) 253:66–72. doi: 10.2460/javma.253.1.66, PMID: 29911947

[30] Woodbridge NT Baines EA Baines SJ. Otitis media in five cats associated with soft palate abnormalities. Vet Rec. (2012) 171:124–5. doi: 10.1136/vr.100720 22798343

[31] Töpfer T Köhler C Rösch S Oechtering G. Brachycephaly in French bulldogs and pugs is associated with narrow ear canals. Vet Dermatol. (2022) 33:214–e60. doi: 10.1111/vde.13067, PMID: 35293639

[32] Nemec A Daniaux L Johnson E Peralta S Verstraete FJ. Craniomaxillofacial abnormalities in dogs with congenital palatal defects: computed tomographic findings. Vet Surg. (2015) 44:417–22. doi: 10.1111/j.1532-950X.2014.12129.x, PMID: 24433432

[33] Perego R Proverbio D Bagnagatti De Giorgi G Della Pepa A Spada E. Prevalence of otitis externa in stray cats in northern Italy. J Feline Med Surg. (2014) 16:483–90. doi: 10.1177/1098612X13512119, PMID: 24226755 PMC11112181

[34] Terziev G Borissov I. Prevalence of ear diseases in dogs – a retrospective 5-year clinical study. Bulg J Vet Med. (2018) 21:76–85. doi: 10.15547/bjvm.1075

[35] O'Neill DG Volk AV Soares T Church DB Brodbelt DC Pegram C. Frequency and predisposing factors for canine otitis externa in the UK - a primary veterinary care epidemiological view. Canine Med Genet. (2021) 8:7. doi: 10.1186/s40575-021-00106-1, PMID: 34488894 PMC8422687

[36] Pye C. *Pseudomonas* otitis externa in dogs. Can Vet J. (2018) 59:1231–4. 30410185 PMC6190182

[37] Bollez A de Rooster H Furcas A Vandenabeele S. Prevalence of external ear disorders in Belgian stray cats. J Feline Med Surg. (2018) 20:149–54. doi: 10.1177/1098612X17700808, PMID: 28375041 PMC11129264

[38] Brame B Cain C. Chronic otitis in cats: clinical management of primary, predisposing and perpetuating factors. J Feline Med Surg. (2021) 23:433–46. doi: 10.1177/1098612X211007072, PMID: 33896249 PMC10741284

[39] Lommer MJ Verstraete FJ. Prevalence of odontoclastic resorption lesions and periapical radiographic lucencies in cats: 265 cases (1995-1998). J Am Vet Med Assoc. (2000) 217:1866–9. doi: 10.2460/javma.2000.217.1866, PMID: 11132894

[40] Cohen-Mivtach E. Prevalence of tooth Resorptive lesions in 120 feline dental patients in Israel. J Vet Dent. (2024) 42:114–7. doi: 10.1177/08987564231226082, PMID: 38295354

[41] Eriksson J Denwood M Nielsen SS McEvoy F Allberg C Thuesen IS. Accuracy of three diagnostic tests to detect tooth resorption in unowned unsocialized cats in Denmark. J Small Anim Pract. (2024) 65:387–93. doi: 10.1111/jsap.13703, PMID: 38234230

[42] Schlicksup MD Van Winkle TJ Holt DE. Prevalence of clinical abnormalities in cats found to have nonneoplastic middle ear disease at necropsy: 59 cases (1991-2007). J Am Vet Med Assoc. (2009) 235:841–3. doi: 10.2460/javma.235.7.841, PMID: 19793014

[43] Sula MM Njaa BL Payton ME. Histologic characterization of the cat middle ear: in sickness and in health. Vet Pathol. (2014) 51:951–67. doi: 10.1177/0300985813511125, PMID: 24280942

[44] Roza MR Silva LA Barriviera M Januario AL Bezerra AC Fioravanti MC. Cone beam computed tomography and intraoral radiography for diagnosis of dental abnormalities in dogs and cats. J Vet Sci. (2011) 12:387–92. doi: 10.4142/jvs.2011.12.4.387, PMID: 22122905 PMC3232399

